# The *FADS1* genotypes modify the effect of linoleic acid-enriched diet on adipose tissue inflammation via pro-inflammatory eicosanoid metabolism

**DOI:** 10.1007/s00394-022-02922-y

**Published:** 2022-06-14

**Authors:** Maija Vaittinen, Maria A. Lankinen, Pirjo Käkelä, Jyrki Ågren, Craig E. Wheelock, Markku Laakso, Ursula Schwab, Jussi Pihlajamäki

**Affiliations:** 1 Institute of Public Health and Clinical Nutrition, University of Eastern Finland, 70210 Kuopio, Finland; 2 Department of Surgery, University of Eastern Finland and Kuopio University Hospital, Kuopio, Finland; 3 Institute of Biomedicine, School of Medicine, University of Eastern Finland, Kuopio, Finland; 4 Division of Physiological Chemistry 2, Department of Medical Biochemistry and Biophysics, Karolinska Institute, Stockholm, Sweden; 5 Institute of Clinical Medicine, Internal Medicine, University of Eastern Finland, Kuopio, Finland; 6 Department of Medicine, Kuopio University Hospital, Kuopio, Finland; 7 Department of Medicine, Endocrinology, and Clinical Nutrition, Kuopio University Hospital, Kuopio, Finland

**Keywords:** Adipose tissue, Arachidonic acid, Eicosanoid, FADS1, Inflammation, Linoleic acid

## Abstract

**Purpose:**

Fatty acid desaturase (*FADS*) variants associate with fatty acid (FA) and adipose tissue (AT) metabolism and inflammation. Thus, the role of *FADS1* variants in the regulation of dietary linoleic acid (LA)-induced effects on AT inflammation was investigated.

**Methods:**

Subjects homozygotes for the TT and CC genotypes of the *FADS1*-rs174550 (TT, *n* = 25 and CC, *n* = 28) or -rs174547 (TT, *n* = 42 and CC, *n* = 28), were either recruited from the METabolic Syndrome In Men cohort to participate in an intervention with LA-enriched diet (FADSDIET) or from the Kuopio Obesity Surgery (KOBS) study. GC and LC–MS for plasma FA proportions and eicosanoid concentrations and AT gene expression for AT inflammatory score (AT-InSc) was determined.

**Results:**

We observed a diet-genotype interaction between LA-enriched diet and AT-InSc in the FADSDIET. In the KOBS study, interleukin (IL)1 beta mRNA expression in AT was increased in subjects with the TT genotype and highest LA proportion. In the FADSDIET, n-6/LA proportions correlated positively with AT-InSc in those with the TT genotype but not with the CC genotype after LA-enriched diet. Specifically, LA- and AA-derived pro-inflammatory eicosanoids related to CYP450/sEH-pathways correlated positively with AT-InSc in those with the TT genotype, whereas in those with the CC genotype, the negative correlations between pro-inflammatory eicosanoids and AT-InSc related to COX/LOX-pathways.

**Conclusions:**

LA-enriched diet increases inflammatory AT gene expression in subjects with the TT genotype, while CC genotype could play a protective role against LA-induced AT inflammation. Overall, the *FADS1* variant could modify the dietary LA-induced effects on AT inflammation through the differential biosynthesis of AA-derived eicosanoids.

**Supplementary Information:**

The online version contains supplementary material available at 10.1007/s00394-022-02922-y.

## Introduction

Dietary fatty acids (FA) have important roles in the regulation of adipose tissue (AT) metabolism, gene expression and inflammation [1, 2]. The dietary intake of the essential omega-6 (n-6) polyunsaturated fatty acid (PUFA) linoleic acid (LA) associates with reduced inflammation [3, 4] whereas arachidonic acid (AA) intake has been shown to associate with increased inflammation [5, 6]. This is suggested to be due to the fact, that AA is a major substrate for pro-inflammatory eicosanoid lipid mediators [7, 8]. Free LA and AA, released from cell membrane by the phospholipase A2 (PLA2), are substrates for the eicosanoid biosynthetic enzymes, including cyclooxygenases (COX), lipoxygenases (LOX), cytochrome P450s (CYP450) and soluble epoxide hydrolase (sEH) [9]. The COX-pathway is associated with the production of pro-inflammatory eicosanoids while eicosanoids produced by some LOXs and CYP450/sEHs are anti-inflammatory [10–12] which could explain why the increased intake of LA or AA has been associated both with increased and reduced inflammation [13].

Two key enzymes responsible for the production of n-6 PUFA AA are delta-5 (D5D) and delta-6 desaturases (D6D), encoded by the fatty acid desaturase (*FADS) 1/2* genes, respectively [14–16]. Recent studies suggest that genetic variations in the *FADS* genes, that reside in near complete linkage disequilibrium in the same locus, associate with glucose homeostasis, PUFA biosynthesis and the proportions of PUFAs in plasma, serum pro-inflammatory markers, hepatic lipid accumulation and inflammation, dyslipidemias [14, 15, 17–19], lipid mediators [18, 20, 21] and also with the risk of type 2 diabetes (T2D) [22].

Previous studies by others and us have shown that the health outcomes of dietary LA intake are regulated by the *FADS1* variants [23–26]. Dietary LA intake has shown to modify the association between *FADS1* variants and clinical phenotypes, including HDL and anthropometric measures [23, 24]. Additionally, we recently demonstrated in a dietary intervention study that the effect of dietary LA intake on serum high-sensitive C-reactive protein (hs-CRP) concentrations depended on the *FADS1*-rs174550 variant (FADSDIET study) [25]. In another study, we showed that AT inflammation correlates positively with the plasma and AT proportion of AA and estimated D6D enzyme activity [27]. This correlation was modified by the *FADS1/2* genetic variation in obese individuals participating in the Kuopio Obesity Surgery (KOBS) study [27]. This suggests that the variants of *FADS* genes could regulate the effect of dietary n-6 PUFA on AT inflammation. However, the effect of dietary LA intake in the association between *FADS1* variant (rs174550) and AT inflammation is poorly understood. In this study, we aimed to clarify the role of *FADS1*-rs174550 in the interaction of dietary LA intake and eicosanoids with AT inflammation. Thus, we investigated in the FADSDIET study (1) if the dietary LA-enriched diet modifies the expression of inflammatory genes in AT and (2) if the regulation of plasma eicosanoid and the expression of inflammatory genes in AT differs between subjects homozygous (CC vs. TT) for *FADS1*-rs174550 variant.

## Experimental section

### Subjects: FADSDIET

The study design of the FADSDIET intervention (NCT02543216) has been previously described [25]. Briefly, 255 healthy normal weight or borderline overweight men homozygous for the *FADS1*-rs174550 (minor allele frequency 0.2977 [28]) were invited from the METabolic Syndrome In Men (METSIM) study to participate in the FADSDIET intervention study. The subjects using an anticoagulant treatment and having severe chronic diseases were excluded from the study. Altogether, 62 men participated in the intervention and 59 of them completed the intervention. AT biopsies (major TT: *n* = 25, minor CC: *n* = 28) and blood samples were taken after overnight fasting at the end of the run-in period (baseline: 0 week) and after LA-enriched dietary intervention (4 week).

#### Dosage information

During the 4-week run-in period they consumed habitual diet but stopped consumption of oil supplements, e.g. fish oil supplements, or plant stanol or sterol enriched products, which were not allowed during the study. During the 4-week intervention period, participants consumed their habitual diet with supplemental LA 30, 40, or 50 mL (27–45 g) in the form of sunflower oil (62% of LA) daily depending on BMI with the increase of ~ 6% of energy intake.

### Subjects: KOBS

Patients accepted to obesity surgery at the Kuopio University Hospital have been recruited to the ongoing Kuopio Obesity Surgery (KOBS) study since 2005 [29]. The present analysis contains cross‐sectional baseline data from a sub-cohort of 70 individuals (males: *n* = 23 and females: *n* = 47), having *FADS1*-rs174547 variant (minor allele frequency 0.2979 [28]) genotyped (only CC: *n* = 28 and TT: *n* = 42) genotypes included in the analysis), gene expression data available from AT and plasma FA composition (clinical characteristics presented in Supplementary Table S1). Furthermore, the *FADS1*-rs174547 is in near complete linkage disequilibrium with the *FADS1*-rs174550.

### Genotyping and gene expression

The study participants were genotyped for the *FADS1* variants rs174550 (FADSDIET) and rs174547 (KOBS), that are in complete linkage disequilibrium, using the TaqMan SNP Genotyping Assay according to the protocol provided by the manufacturer (Applied Biosystems, Foster City, CA, USA). For the gene expression, AT samples were immediately frozen in liquid nitrogen. TruSeq Targeted RNA Expression (TREx) platform with the MiSeq system (Illumina, San Diego, CA) was used for measuring gene expression levels in AT, as previously described [27, 30]. Additionally, the interleukin 1 beta (*IL1β*) gene expression in obese subjects (*n* = 70) of the KOBS study was measured, as described previously [30]. To investigate the effect of dietary LA intake on AT inflammation and whether the *FADS1*-rs174550 could modify this interaction, we measured the expression of genes which are increased in the AT of subjects with obesity and have a major role in the development of insulin resistance, and T2D (specific associations with inflammation are shown in Supplementary Table S2 with references).

### Biochemical analyses (lipids, glucose, insulin, FA and lipid mediator profiling)

Commercial kits (Thermo Fisher Scientific) were used for measuring concentrations of serum total, LDL, and HDL cholesterol and total triglycerides. The Konelab 20XTi Clinical Chemistry Analyzer (Thermo Fisher Scientific) and Enzymatic photometric (glucose hexokinase) method (Thermo Fisher Scientific) was used for the measurement of plasma glucose concentration. Chemiluminometric immunoassay method (DiaSorin Liaison Analyzer; DiaSorin GmbH) was used for the analysis of plasma insulin concentration.

FA composition in plasma lipid fractions was analyzed according to previously described method [30]. Briefly, plasma samples were extracted with chloroform–methanol (2:1), and the different lipid fractions, cholesteryl esters (CE), triglycerides (TG), and phospholipids (PL) were separated by solid phase extraction with an aminopropyl column. Samples were transmethylated with 14% boron trifluoride in methanol and were analyzed by 7890A gas chromatograph (Agilent Technologies, Inc., Wilmington, DE, USA) equipped with a 25‐m FFAP column. Cholesteryl nonadecanoate (Nu Chek Prep, Inc., Elysian, MA, USA), trinonadecanoin, and phosphatidylcholine dinonadecanoyl (Larodan Fine Chemicals, Malmö, Sweden) served as internal standards. Enzyme activities in different lipid fractions were estimated as product-to-precursor ratios of individual FAs as follows: D5D = 20:4 n-6/20:3 n-6 and D6D = 18:3 n-6/18:2 n-6. Total n-6 PUFA and total n-3 PUFA composition was calculated as a sum of corresponding individual FA proportion. Plasma PL fraction was chosen due to its rapid response to dietary changes in fat, and the fact that CEs are produced by the transfer of FAs from PLs [31]. Thus, PL fraction was chosen for further analyses. Lipid mediator profiling in plasma was performed by liquid chromatography–mass spectrometry, as previously described [32].

### Statistics

Statistical analyses were conducted with the SPSS software (version 25, IBM Corp., Armonk, NY, USA) and GraphPad Prism 5.03 for Windows (GraphPad Software, San Diego, CA, USA). Independent sample’s *t* test was used to compare differences between the genotypes. Within group differences between the two timepoints were analyzed with paired sample’s *t* test. Repeated-measures general linear model (GLM) was used for the assessment of diet-genotype interaction. One-way analysis of variance (ANOVA) with Bonferroni’s multiple comparison test was used for testing differences in plasma PL-LA tertiles in different genotype groups of the *FADS1*-rs174547 in the KOBS study. The mRNA expression data of inflammatory genes in AT were normalized using *Z*-transformation, *Z*-score, which is a standardized variable with standard deviation of one and a mean of zero. Next, a combined inflammation *Z*-score was created by calculating the average from the individual *Z*-scores of inflammatory genes in AT. Combining inflammatory genes using a *Z*-score provides a more accurate characterization of inflammatory status in tissue [33]. Furthermore, the mean *Z*-score of individual serum inflammatory factors has been previously used as a surrogate marker for low-grade inflammation [33, 34]. A Shapiro–Wilk’s test was used to examine the normal distribution of variables and the variables were logarithmically (log) transformed when needed. Correlations between the variables were analyzed either using partial correlations adjusted for age for log-transformed variables or using Spearman’s nonparametric correlation. For the grouped correlation analyses regarding eicosanoids, the Spearman’s correlation coefficients were transformed using Fisher's *r* to *z* transformation and standard error of correlation was calculated ($\sqrt{(1-r)^ \wedge 2}/\sqrt{(n-2)}$), where *r* is correlation coefficient and *n* is the number of samples. The *z*-transformed results of the Spearman’s correlation analysis between AT-InSc and individual eicosanoid concentration in plasma were pooled and the deviation of the average Spearman’s correlations between AT-InSc and eicosanoid parameters from the null hypothesis (correlation coefficient = 0) was tested using one sample *t* test. Additionally, the *z*-transformed correlations were used for analyzing the differences between the two timepoints (baseline vs. follow-up) within the genotype group by using paired samples *t* test. In the correlation analyses, we report significant results both with and without Bonferroni correction for multiple testing. For the TREx analysis, the expression levels for each gene per sample in the gene panel were normalized based on the total number of aligned reads of the corresponding sample. The results are shown as percentage of total transcript reads. Descriptive statistics are presented as mean ± SD or SEM (indicated in table and figures) and *p* ≤ 0.05 was considered as statistically significant.

## Results

### Clinical characteristics

The results of clinical characteristics in the whole study population (TT: *n* = 26, CC: *n* = 33) of the FADSDIET intervention have been published previously, and the adherence to the consumption of sunflower oil was excellent [25]. The parameters for participants from which AT biopsies were available (TT: *n* = 25, CC: *n* = 28) are shown in Table 1. The only significant difference at baseline was that the participants with the CC genotype were older than those with the TT genotype (CC: 58.8 ± 3.1 years vs TT: 55.1 ± 2.2 years, *p* < 0.0001). Fasting plasma glucose concentration decreased in participants with the CC genotype (paired-samples *t* test, *p* = 0.01; diet-genotype interaction, *p* = 0.012) but not in participants with the TT genotype during the intervention (Table 1). Additionally, serum total cholesterol concentration decreased in participants with the CC genotype (*p* = 0.017) but not in those with TT genotype. Serum LDL cholesterol concentration decreased in both genotype groups (*p* = 0.011, *p* = 0.010) during the intervention. There were no differences in the clinical variables between the genotypes of *FADS1*-rs174547 in the KOBS study (Supplementary Table S1).

**Table 1:** Clinical characteristics of the FADSDIET participants according to the *FADS1*-rs174550 variant at baseline and after LA-enriched diet

	0 week		4 weeks		*p*^a^	*p*^b^		*p*^c^
	TT (*n* = 25)	CC (*n* = 28)	TT (*n* = 25)	CC (*n* = 28)		TT	CC	
Age, years	55.1 ± 2.2	58.8 ± 3.1			**7 × 10**^**–5**^			
Weight, kg	80.9 ± 10.5	78.7 ± 9.2	80.9 ± 10.5	78.7 ± 9.1	0.416	0.981	0.713	0.828
BMI, kg/m^2^	25.6 ± 2.4	24.6 ± 2.3	25.7 ± 2.4	24.6 ± 2.3	0.101	0.817	0.735	0.972
Systolic blood pressure, mmHg	128 ± 12	129 ± 13	126 ± 9	127 ± 13	0.749	0.207	0.061	0.986
Diastolic blood pressure, mmHg	82 ± 7	84 ± 8	82 ± 7	82 ± 8	0.486	0.878	0.066	0.188
Waist circumference, cm	94.4 ± 8.5	93.0 ± 6.9	94.3 ± 8.5	92.9 ± 6.8	0.532	0.513	0.235	0.631
Plasma glucose^d^, mmol/L	5.6 ± 0.4	5.7 ± 0.3	5.6 ± 0.5	5.6 ± 0.3	0.144	0.385	**0.007**	**0.012**
Plasma insulin^d^, mU/L	7.4 ± 3.2	7.9 ± 3.5	7.8 ± 3.1	7.9 ± 4.4	0.578	0.500	0.991	0.651
Serum total cholesterol^d^, mmol/L	5.2 ± 0.8	5.4 ± 1.2	5.0 ± 0.6	5.1 ± 1.0	0.506	0.156	**0.017**	0.451
Serum HDL cholesterol^d^, mmol/L	1.4 ± 0.4	1.4 ± 0.3	1.4 ± 0.4	1.4 ± 0.3	0.580	0.439	0.531	0.878
Serum LDL cholesterol^d^, mmol/L	3.2 ± 0.6	3.3 ± 1.0	3.0 ± 0.5	3.0 ± 0.8	0.663	**0.011**	**0.010**	0.741
Serum triglycerides^d^, mmol/L	1.2 ± 0.6	1.4 ± 0.8	1.3 ± 0.7	1.5 ± 1.1	0.184	0.298	0.309	0.856
Plasma free fatty acids^d^, mmol/L	0.4 ± 0.1	0.5 ± 0.1	0.5 ± 0.2	0.5 ± 0.2	0.196	0.223	0.620	0.213

Values are means ± SDs. Bold values are statistically significant

*FADS1* fatty acid desaturase 1, *LA* linoleic acid

^a^Independent samples *t* test for differences between genotypes at baseline

^b^Paired-samples *t* test for the change within the genotype

^c^Diet-genotype interaction, repeated-measures general linear model

^d^Fasting

### *FADS1*-rs174550 modifies the effect of LA intake on AT inflammation

**Fig. 1:**
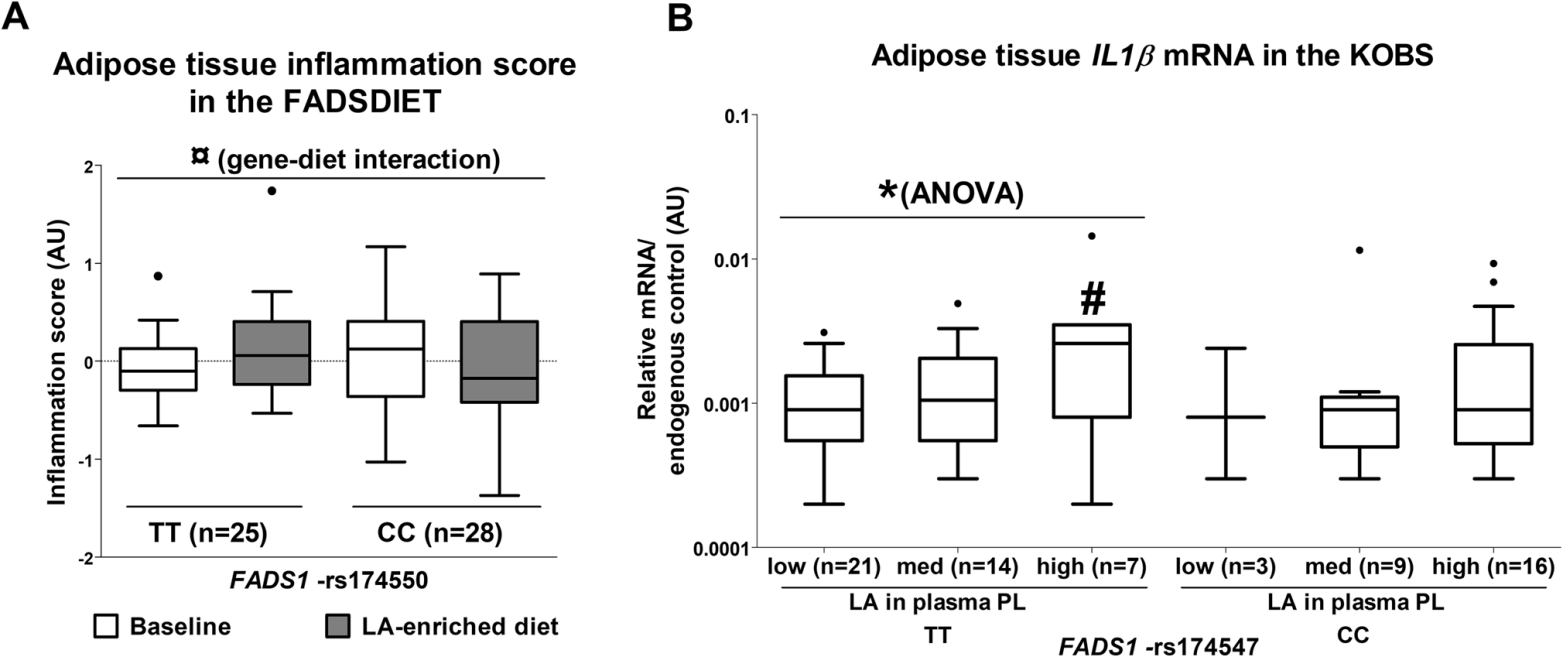
The effect of linoleic acid (LA) on adipose tissue inflammation. **A** The effect of diet enriched with linoleic acid (LA) on adipose tissue inflammatory score in the FADSDIET study and **B** the association of LA tertiles of plasma phospholipid (PL) fraction on adipose tissue *IL1β* expression in the KOBS study, according to the *FADS1* variants rs174550 and rs174547, respectively. Values are presented as means ± SEM. Black circles, outliers, ¤*p* < 0.05 (GLM repeated measures), *p < 0.05 (GLM ANOVA), #*p* < 0.05 (GLM ANOVA, pairwise Bonferroni’s multiple comparison between the lowest and highest LA proportion in plasma PL fraction)

The results showed a significant diet-genotype interaction (repeated-measures GLM) between dietary LA-enriched diet and AT-InSc (*p* = 0.019, Fig. 1A) in the FADSDIET. There was a non-significant trend towards a reduced AT-InSc in subjects with the CC genotype (*p* = 0.067), while the AT-InSc did not change in subjects with the TT genotype (*p* = 0.130) of the *FADS1*-rs174550 in response to a LA-enriched diet (Fig. 1A). In line with this, we could additionally demonstrate that *IL-1β* mRNA expression in subcutaneous AT was statistically different between the groups of LA proportion in plasma PL fraction in subjects with the TT genotype (ANOVA, *p* = 0.019) (Fig. 1B). After adjustment for Bonferroni’s multiple comparison, obese subjects with the highest LA proportion in plasma PL fraction had increased *IL-1β* expression in subcutaneous AT when compared to the group with the lowest LA proportion in plasma PL fraction in subjects with the TT genotype (*p* = 0.017) but not in those with the other genotype (CC) of *FADS1*-rs174547 at baseline of the KOBS study (Fig. 1B).

### AT-InSc correlates differently with plasma n-6 and LA proportions and eicosanoid levels between individuals with the different *FADS1* genotypes

#### Correlations between AT-InSc and n-6 and LA proportions in plasma PL fraction

**Fig. 2:**
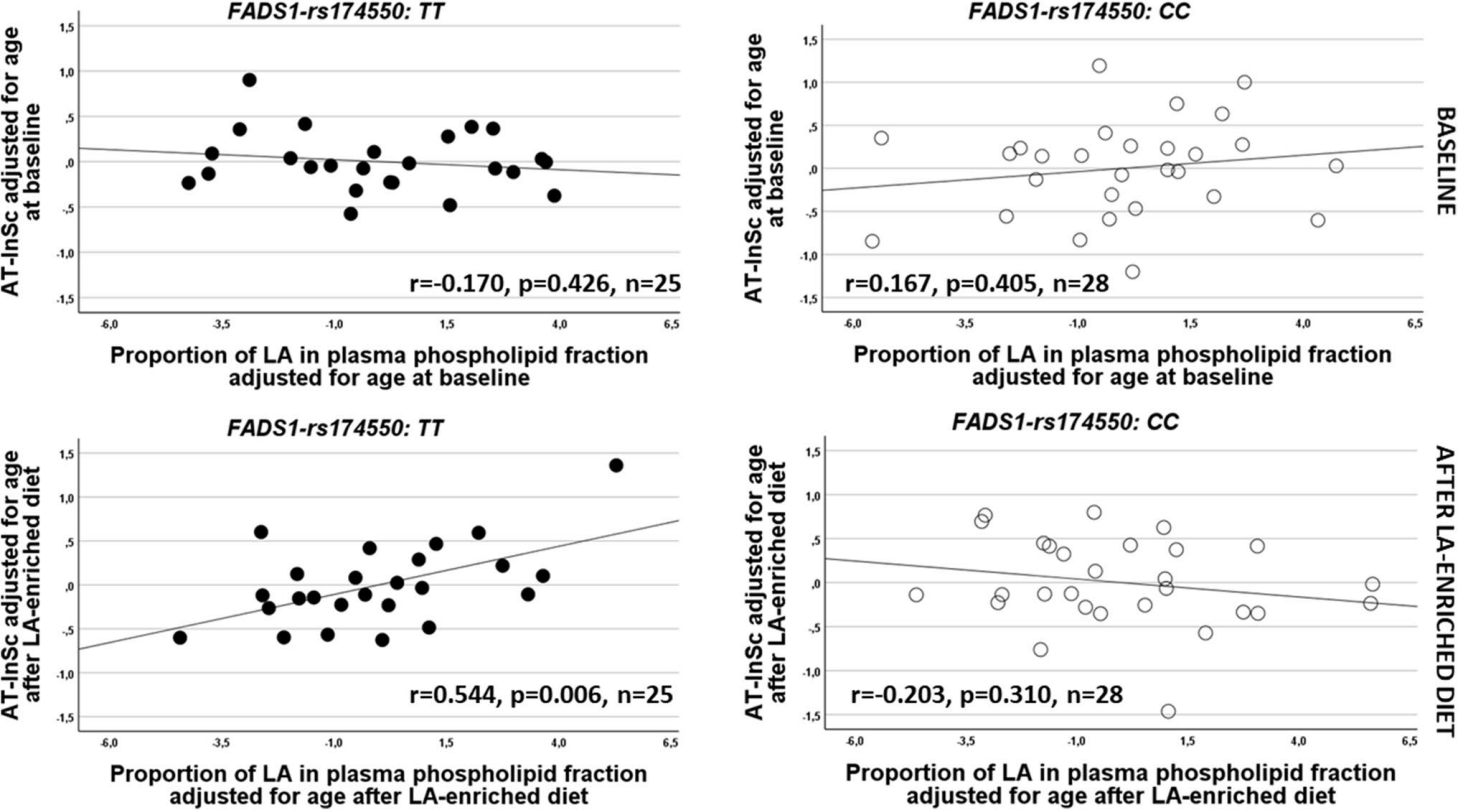
Correlations between adipose tissue inflammation and linoleic acid (LA) proportion in plasma phospholipid (PL) fraction. Scatterplots demonstrating the Pearson’s partial correlation coefficients adjusted for age between adipose tissue inflammatory score (AT-InSc) and LA proportion in plasma PL fraction (log-transformed) at baseline and after LA-enriched diet according to the fatty acid desaturase 1 (*FADS1*)-rs174550 genotypes. *r* Pearson’s correlation coefficient adjusted for age, *p* *p* value, *n* sample size, *PL* phospholipid; black circles, TT genotype; open circles, CC genotype

Since there was a difference in age between the *FADS1* genotypes at baseline, the correlations between AT-InSc and plasma FA proportions in PL fraction were adjusted for age. The results showed that AT-InSc correlated negatively with total n-6 PUFA proportion in plasma PL fraction at baseline (*r* = − 0.449, *p* = 0.028) but positively after LA-enriched diet (*r* = 0.494, *p* = 0.014) in subjects with the TT genotype of the *FADS1*-rs174550 (Supplementary Table S3). Additionally, AT-InSc correlated positively with the proportion of LA in plasma PL fraction after LA-enriched diet (*r* = 0.544, *p* = 0.006) in subjects with the TT genotype of the *FADS1*-rs174550 (Fig. 2, Supplementary Table S3). In contrast, there were no significant correlations in subjects with the CC genotype of the *FADS1*-rs174550 between AT-InSc and n-6 or LA (Fig. 2, Supplementary Table S3). The correlation results between AT-InSc and other individual FA proportions in plasma PL fraction are shown in Supplementary Table S3.

#### Correlations between AT-InSc and eicosanoids

**Fig. 3:**
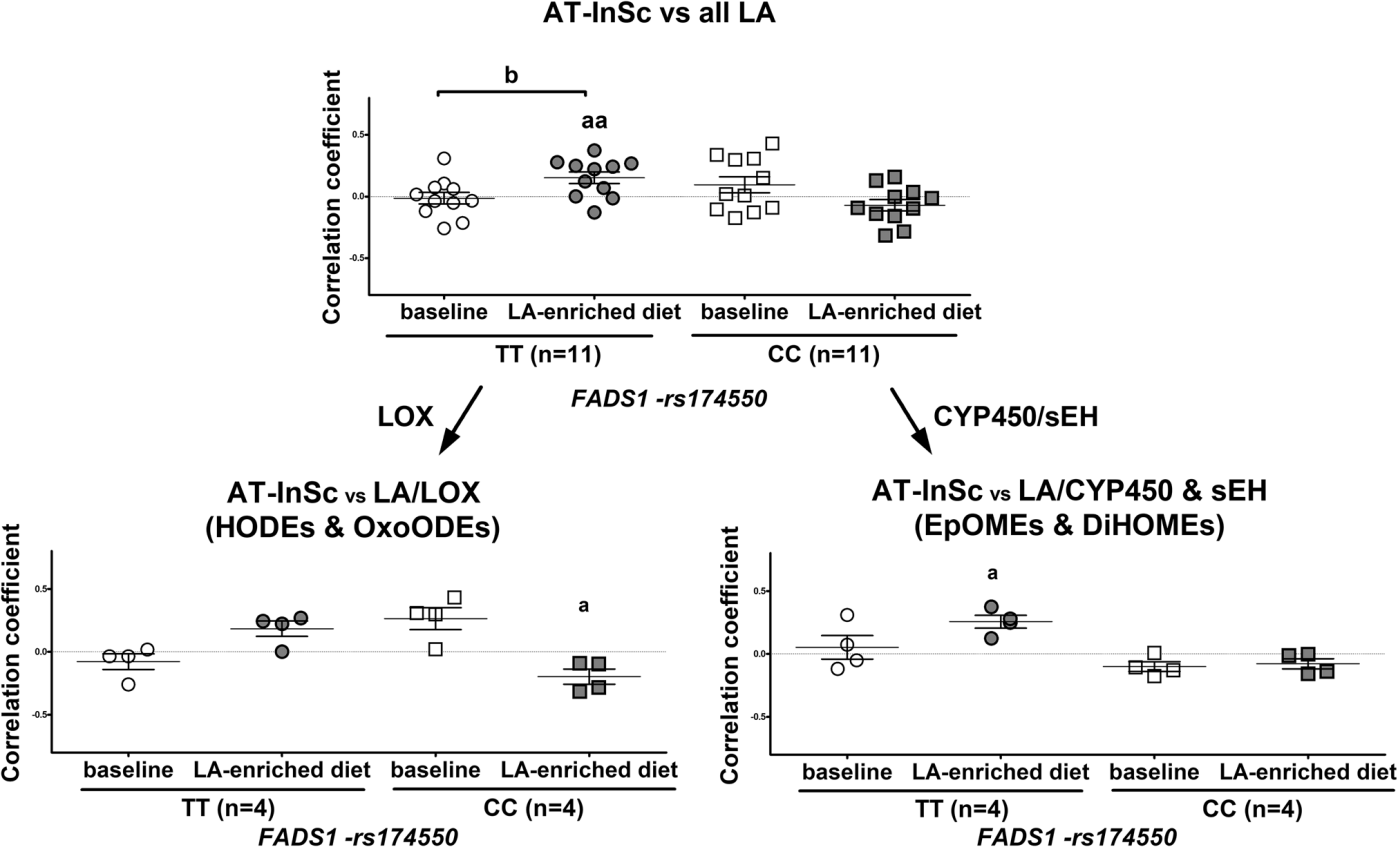
Correlations between adipose tissue inflammation and linoleic acid (LA)-derived eicosanoids in plasma. Spearman’s correlation coefficients of adipose tissue inflammatory score (AT-InSc) with the average of all LA-derived eicosanoids (*n* = 11), and the groups of eicosanoids mediated via different pathways, including LOX (*n* = 4) and CYP450/sEH (*n* = 4) in the FADSDIET study divided by the *FADS1*-rs174550. LA, linoleic acid; LOX, lipoxygenases; CYP, cytochrome P450; sEH, soluble epoxide hydrolase; EpOMEs, epoxy-octadecenoic acids; DiHOMEs, dihydroxy-octadecenoic acids; HODE, hydroxyoctadecadienoic acids; OxoODEs, oxo-octadecadienoic acids. ^a^*p* < 0.05, ^aa^*p* < 0.01, ^aaa^*p* < 0.001 (one sample *t* test); ^b^*p* < 0.05, ^bb^*p* < 0.01, ^bbb^*p* < 0.001 (paired samples *t* test)

**Fig. 4:**
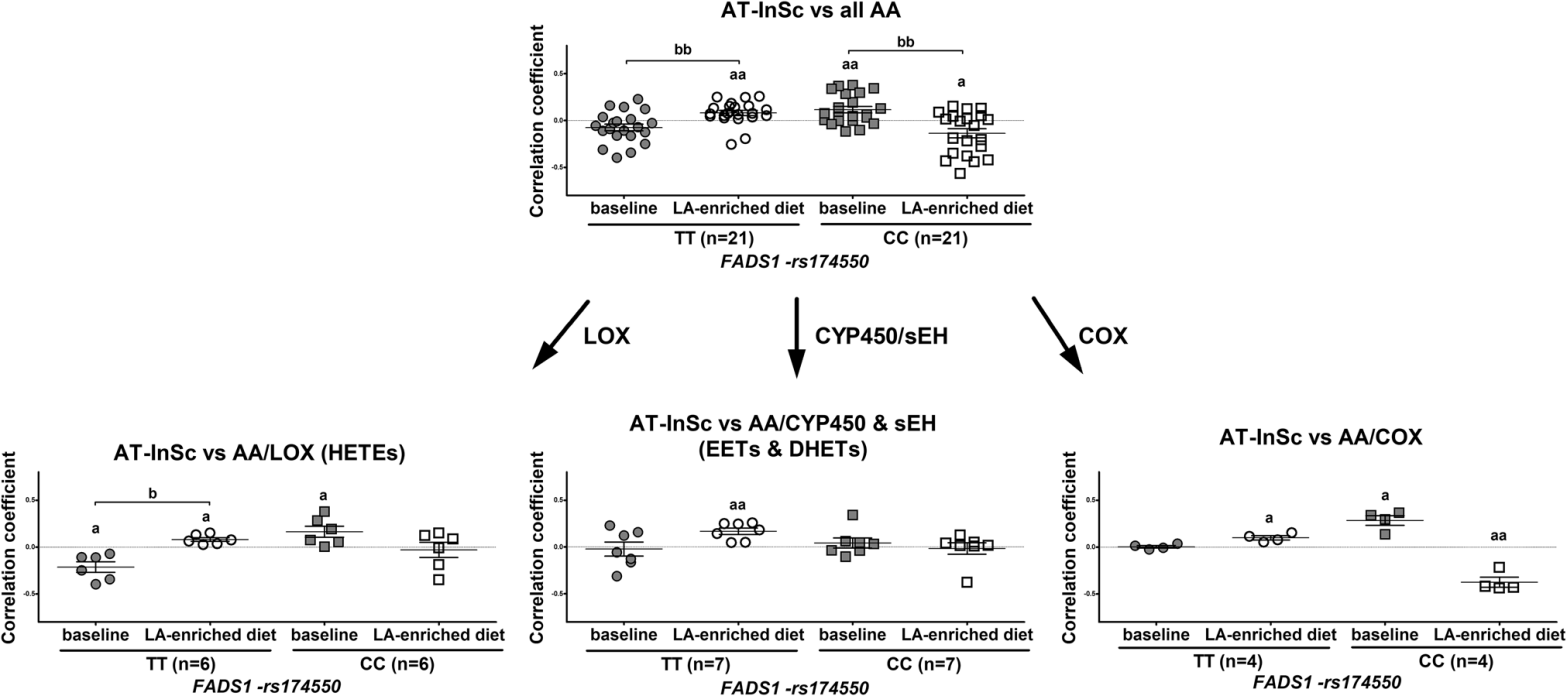
Correlations between adipose tissue inflammation and arachidonic acid (AA)-derived eicosanoids in plasma. Spearman’s correlation coefficients of adipose tissue inflammatory score (AT-InSc) with the average of all AA-derived eicosanoids (*n* = 21), and the groups of eicosanoids mediated via different pathways, including LOX (*n* = 6), CYP450/sEH (*n* = 7), and COX (*n* = 4) in the FADSDIET study divided by the *FADS1*-rs174550. *AA* arachidonic acid, *LOX* lipoxygenases, *COX* cyclooxygenase, *CYP* cytochrome P450, *sEH* soluble epoxide hydrolase, *EETs* epoxyeicosatrienoic acids, *DHETs* dihydroxy-eicosatrienoic acids. ^a^*p* < 0.05, ^aa^*p* < 0.01, ^aaa^*p* < 0.001 (one sample *t* test); ^b^*p* < 0.05, ^bb^*p* < 0.01, ^bbb^*p* < 0.001 (paired samples *t* test)

We investigated whether correlations between AT-InSc and plasma LA- or AA-derived eicosanoid compositions differed between the subjects with TT and CC genotypes. There was on average a positive correlation (*r* = 0.153, *p* = 0.008) between the AT-InSc and all LA-derived eicosanoids at the end of the intervention in subjects with the TT genotype of the *FADS1*-rs174550 (Fig. 3). There was also a significant difference in the correlation between the AT-InSc and LA-derived lipid mediators between the two timepoints (baseline vs. the end of the intervention) within TT genotype (*p* = 0.024), but not within CC genotype, of the *FADS1*-rs174550 (Fig. 3). There was on average a positive correlation (*r* = 0.081, *p* = 0.008) between AT-InSc and all AA-derived eicosanoids at the end of the intervention in subjects with the TT genotype of the *FADS1*-rs174550 (Fig. 4). In contrast, there was a positive correlation at baseline (*r* = 0.116, *p* = 0.003, corrected for multiple comparison *p* = 0.042) and a negative correlation (*r* = − 0.138, *p* = 0.011) at the end of the intervention between AT-InSc and all AA-derived eicosanoids in subjects with the CC genotype (Fig. 4). Moreover, there was a significant difference in the correlation between the AT-InSc and AA-derived eicosanoids between the two timepoints (baseline vs. the end of the intervention) within TT genotype (*p* = 0.078) and within CC genotype (*p* = 0.010), of the *FADS1*-rs174550 (Fig. 4).

Next, we explored whether there are differences in the mean correlations between AT-InSc and eicosanoids synthesized via COX, LOX, CYP450/sEH pathways between the individuals with the different *FADS1* genotypes. This mean correlation summarizes the overall direction of correlation related to the specific pathway. LA-derived eicosanoids related to the CYP450/sEH-pathway correlated positively (*r* = 0.257, *p* = 0.016) with the AT-InSc at the end of the intervention in subjects with the TT, but not with the CC genotype of *FADS1*-rs174550 (Fig. 3). On the other hand, LA-derived eicosanoids related to the LOX-pathway correlated negatively (*r* = − 0.197, *p* = 0.046) with the AT-InSc at the end of the intervention in subjects with the CC, but not with the TT genotype of *FADS1*-rs174550 (Fig. 3). Furthermore, AA-derived eicosanoids related to the CYP450/sEH-pathway correlated positively (*r* = 0.168, *p* = 0.003) with the AT-InSc at the end of the intervention in subjects with the TT, but not with the CC genotype of *FADS1*-rs174550 (Fig. 4). Additionally, AA-derived eicosanoids related to the LOX-pathway correlated negatively at baseline but positively at the end of intervention (*r* = − 0.214, *p* = 0.012; *r* = 0.081, *p* = 0.011, respectively) with the AT-InSc in subjects with the TT genotype. Moreover, the correlation between AT-InSc and eicosanoids related to the LOX-pathway differed significantly between the two timepoints (baseline vs. the end of the intervention) within TT genotype (*p* = 0.031), but not within CC genotype, of the *FADS1*-rs174550 (Fig. 4). There was also a positive correlation (*r* = 0.164, *p* = 0.040) between the AT-InSc and eicosanoids related to the LOX-pathway at baseline in subjects with the CC genotype (Fig. 4). Eicosanoids related to COX-pathway correlated positively (*r* = 0.099, *p* = 0.021) with the AT-InSc at the end of intervention in subjects the TT genotype. In contrast, eicosanoids related to the COX-pathway correlated positively at baseline but negatively at the end of intervention (*r* = 0.284, *p* = 0.012; *r* = − 0.377, *p* = 0.006, respectively) with the AT-InSc in subjects with the CC genotype.

## Discussion

We observed that the *FADS1*-rs174550 variant modifies the effect of dietary LA intake on AT inflammation in normal/overweight subjects in our study (*p* = 0.019 for gene-diet interaction). We found an association between an increased proportion of LA in plasma PL fraction, reflecting dietary LA intake, and AT inflammation in individuals with obesity with the TT, but not with the CC genotype in the KOBS study. Correlation analyses demonstrated that there was a genotype-dependent difference between LA proportions in plasma PL fraction and AT inflammation after LA-enriched diet. Interestingly, the correlations between LA- or AA-derived eicosanoids and AT inflammation differed between the participants homozygous (CC vs. TT) for the *FADS1*-rs174550 variant, suggesting that the *FADS1* genotypes may modify the shuttling of eicosanoid synthesis to the CYP450/sEH, COX and LOX pathways and could partly explain differential responses to LA-enriched diet between the individuals with the *FADS1* genotypes.

Dietary LA intake has shown to associate with a reduced risk for cardiovascular diseases [3]. However, there also are evidence that LA could induce pro-inflammatory events [35, 36]. Our main finding was that dietary LA-enriched diet decreased AT inflammation, as expected based on the known anti-inflammatory effects of LA [3, 4, 37], but only in subjects with the CC genotype of *FADS1*-rs174550 in the FADSDIET study. On the other hand, the AT inflammation was increased in subjects with the TT genotype of *FADS1*-rs174550/rs174547 either in response to LA-enriched diet (FADSDIET) or in individuals in the highest tertile of LA in plasma PL fraction (KOBS). Furthermore, our correlation analyses between LA proportions and AT-InSc demonstrated a positive correlation after LA-enriched diet only in subjects with the TT genotype of *FADS1*-rs174550. Obesity is associated with increased AT inflammation which is considered as major factors for the development of insulin resistance, T2D, and cardiovascular diseases [38]. Thus, the increased AT inflammation after dietary LA-enriched diet may predispose the subjects with the TT genotype of *FADS1*-rs174550 at higher risk for developing dysfunctional AT and insulin resistance. These results-suggest that the effect of dietary LA on AT inflammation depends on the *FADS1* genotype.

Our following analyses demonstrated that the differences between the genotypes could also be related to the differential metabolism of LA- and AA-derived eicosanoids in plasma. The results demonstrated a positive correlation between AT-InSc and the average concentration of all LA- or AA-derived eicosanoids after the LA-enriched diet in subjects with the TT genotype of *FADS1*-rs174550. In contrast, there was a negative correlation between AT-InSc and the average level of all AA-derived eicosanoids after dietary LA intake in subjects with the CC genotype of *FADS1*-rs174550. It is suggested that LA-derived metabolites can mediate inflammation [39, 40], and that AA-derived eicosanoids promote inflammation [10, 12]. Thus, it is possible that high intake of dietary LA and consequent changes in eicosanoid metabolism could contribute to low-grade inflammation in AT depending on the *FADS1* variant.

When investigating further the pathways through which the eicosanoids are synthesized, we could demonstrate that the dietary LA-enriched diet resulted in a positive correlation between AT-InSc and the average of LA- and AA-derived eicosanoids related to the CYP450/sEH-pathway in subjects with the TT genotype, but not the CC genotype of *FADS1*-rs174550. In contrast, there was a negative correlation between AT-InSc and the average of LA-derived eicosanoids related to the LOX-pathway in subjects with the CC genotype; whereas, AA-derived eicosanoids related to the LOX-pathway correlated positively in subjects with the TT genotype of *FADS1*-rs174550 after LA-enriched diet. Additionally, AA-derived eicosanoids related to the COX-pathway correlated positively in subjects with the TT genotype of *FADS1*-rs174550 after LA-enriched diet. Most of the individual lipid mediators included in the analyses have pro-inflammatory effects. For example, the LA-derived lipid mediators of hydroxyoctadecadienoic acids (HODEs, LOX-pathway) are markers of oxidative stress and the regulators of inflammation [41], and epoxy-octadecenoic acids (EpOMEs, CYP450-pathway) and dihydroxy-octadecenoic acids (DiHOMEs, sEH-pathway) can act as protoxins [42], induce oxidative stress and NF-KB and promote inflammation [43, 44]. In addition, many AA-derived eicosanoids, like prostaglandins (COX-pathway), hydroxy-eicosatetraenoic acids (HETEs, LOX pathway) and dihydroxy-eicosatrienoic acids (DHETs, sEH-pathway), are known to induce inflammation [45]. Previous studies have shown that the *FADS1* variation contributes to inflammation via differential enzyme activity and eicosanoid biosynthesis. Hester et al. [20] have shown that the *FADS1* variation could modify LOX-mediated eicosanoid levels in human blood, indicating that *FADS1* genotypes could contribute to inflammation through differential eicosanoid biosynthesis. Additionally, AA-derived pro-inflammatory eicosanoids mediated via LOX- and COX-pathways in intestine have shown to be decreased in a rodent model of *FADS1* null mice, reflecting the decreased *FADS1* function, like the CC genotype of the *FADS1*-rs174550 [21]. Furthermore, Gromovsky et al. [18] have shown that the *FADS1* knockdown induced a differential expression of COX/LOX genes. On the other hand, the *FADS1* overexpression has shown to induce pro-inflammatory prostaglandin E2 (PGE2) level which is mediated by the COX [46]. Taken together, since the *FADS1* has shown to regulate the activity of enzymes or their eicosanoids these results suggest that LA-enriched diet induces AT inflammation in those with the TT genotype, the genotype known to be associated with the T2D [47], possibly via the increased levels of enzymes responsible for the biosynthesis of pro-inflammatory eicosanoids. In contrast, the CC genotype of *FADS1*-rs174550 could have a protective role against dietary LA-induced effects on AT inflammation, and this may be related to the decreased levels of enzymes responsible for the biosynthesis of pro-inflammatory eicosanoids. We fully acknowledge that further studies are needed to clarify the mechanisms underlying these genotype-specific relationships between AT inflammation and LA/AA-related eicosanoid metabolism.

The previous study of our group demonstrated that hs-CRP level in serum was decreased after LA-enriched diet in subjects with the TT genotype, while in subjects with the CC genotype hs-CRP level was increased [25]. The discrepancy in the results between the decreasing effect of LA-enriched diet on hs-CRP in our previous study [25], and the increasing effect on AT inflammation in this study in subjects with the TT genotype could be in part explained by the fact that CRP is mainly synthesized by the liver [48] while in AT, that was the main topic of this study, the regulation may be different. This is in line with our previous observation that AA proportion in plasma/AT correlated positively with AT inflammation but negatively with the liver inflammation in subjects with obesity and the major genotype of *FADS2*-rs174616 (located in the same *FADS* cluster as rs174550 and rs174547) in the KOBS study [27]. Furthermore, a reduced *FADS1* function, due to the CC genotype of *FADS1*-rs174550 with a lower desaturase activity, has been shown to associate positively with hepatic inflammation, non-alcoholic fatty liver disease and to induce changes in n-6 and n-3 PUFA-derived eicosanoid levels in a mouse model and in a human study [17, 18]. These results suggest that the *FADS1*-rs174550 variant could modify the association between PUFA metabolism and inflammation tissue dependently. Unfortunately, it is difficult to obtain serial liver biopsies, unlike AT biopsies, during a dietary intervention.

The strengths of our study are the unique study design with the genotype-based recruitment of individuals [25]. Additionally, the availability of AT biopsies and FA composition in plasma enabled to investigate the genetic influence of *FADS1* in the relationship between n-6 PUFA metabolism and inflammation in AT. Another strength of this study is that we were able to combine results from normal/overweight individuals (FADSDIET) with obese individuals of the cross-sectional KOBS study. Due to the fact, that the subjects were chosen based in their specific *FADS1* variant and were middle-aged men in the FADSDIET, the results cannot be generalized to women or younger population. Additionally, it is well known that gene expression data does not necessarily reflect protein expression or the activity of proteins at the tissue and therefore, the lack of protein data in AT for the inflammatory genes limits the implications of the findings. Unfortunately, protein analysis was not possible due to the sample size limitations in the FADSDIET study. Although, our previous study successfully demonstrated LA-induced changes in the AA proportion in plasma PL fraction according to the *FADS1* variant in the same cohort [25]. It is possible that we may not detect individual dietary LA-induced effects on AT inflammation or eicosanoid levels due to a small sample size. Although the chosen single SNP is unlikely to be causative, the *FADS1*-rs174550 has a high effect size on plasma and tissue FA concentrations and it has been previously used as a marker for the variation in the *FADS1/2* locus [22, 49]. Unfortunately, we were able to determine FA and eicosanoid concentrations only in plasma. Knowledge about the tissue FA levels could give further insights in the interaction between PUFA metabolism and inflammation in AT.

In conclusion, the *FADS1*-rs174550 variant regulates the interaction between dietary LA intake and AT inflammation. Increased dietary LA intake induced a positive relationship between AT inflammation and eicosanoids mediated through CYP450-pathway in subjects with the TT genotype of *FADS1*-rs174550. On the contrary, there was a negative relationship between AT inflammation and eicosanoids mediated through the COX-pathway in subjects with the CC genotype of *FADS1*-rs174550. Our results suggest that CC genotype could play a protective role against LA-induced AT inflammation. Overall, the *FADS1* variant could modify the dietary LA-induced effects on AT inflammation through differential biosynthesis of AA-derived eicosanoids.

## Supplementary Information

Below is the link to the electronic supplementary material.

Supplementary file1 (DOCX 44 kb)
