# Supplementary material for: The FADS1 genotypes modify the effect of linoleic acid-enriched diet on adipose tissue inflammation via pro-inflammatory eicosanoid metabolism
Source: Eur J Nutr. 2022 Jun 14;61(7):3707–18. doi: 10.1007/s00394-022-02922-y (PMC9464166; doi:10.1007/s00394-022-02922-y)
Supplement: Supplementary file 1 — Supplementary file1 (DOCX 44 kb) [file 394_2022_2922_MOESM1_ESM.docx]

The *FADS1* genotype modifies the effect of linoleic acid-enriched diet on adipose tissue inflammation *via* pro-inflammatory eicosanoid metabolism

Maija Vaittinen^1^, Maria Lankinen^1^, Pirjo Käkelä^2^, Jyrki Ågren^3^, Craig E Wheelock^4^, Markku Laakso^5,6^, Ursula Schwab^1,7^, and Jussi Pihlajamaki^1,7^

^1^Institute of Public Health and Clinical Nutrition, University of Eastern Finland, Kuopio, Finland

^2^ Department of Surgery, University of Eastern Finland and Kuopio University Hospital, Kuopio, Finland

^3^ Institute of Biomedicine, School of Medicine, University of Eastern Finland, Kuopio, Finland

^4^Division of Physiological Chemistry 2, Department of Medical Biochemistry and Biophysics, Karolinska Institute, Stockholm, Sweden

^5^ Institute of Clinical Medicine, Internal Medicine, University of Eastern Finland, Kuopio, Finland

^6^ Department of Medicine, University of Eastern Finland, Kuopio, Finland

^7^ Department of Medicine, Endocrinology, and Clinical Nutrition; Kuopio University Hospital, Kuopio, Finland

**Corresponding author:** Maija Vaittinen (PhD), Institute of Public Health and Clinical Nutrition, University of Eastern Finland, 70210 Kuopio, Finland, email: maija.vaittinen@uef.fi

**Supplementary Table S1.** Clinical characteristics at baseline of the KOBS participants according to the genotypes of *FADS1*-rs174547 variant.

|  | **TT (n=42)** | **CC (n=28)** | **p** |  |
| --- | --- | --- | --- | --- |
| BMI, kg/m^2^ | 44.4±6.3 | 43.5±6.3 | 0.587 |  |
| age, years | 47.5±9.1 | 46.9±8.9 | 0.769 |  |
| Plasma glucose^3^, mmol/L | 6.7±1.8 | 6.2±1.2 | 0.252 |  |
| Plasma insulin^3^, mU/L | 18.8±10.9 | 18.3±8.7 | 0.845 |  |
| Serum total cholesterol^3^, mmol/L | 4.4±1.1 | 4.4±1.1 | 0.983 |  |
| Serum HDL cholesterol^3^, mmol/L | 1.1±0.3 | 1.1±0.4 | 0.784 |  |
| Serum LDL cholesterol^3^, mmol/L | 2.6±0.9 | 2.7±1.1 | 0.810 |  |
| Serum triglycerides^3^, mmol/L | 1.5±0.6 | 1.5±0.6 | 0.900 |  |
| Values are means ± SDs. FADS1, fatty acid desaturase 1; BMI, body mass index; HDL, high density lipoprotein; LDL, low density lipoprotein; p=GLM univariate (between genotypes) | | | |  |
|  |  |  |  |  |

**Supplementary Table S2.** The list of analyzed genes in the study. NFKB: nuclear factor kappa-light-chain-enhancer of activated B cells, IR: insulin resistance, T2D: type 2 diabetes

| **GENE NAME** | **GENE SYMBOL** | | **INVOLVED IN** | | **REFERENCE** | |
| --- | --- | --- | --- | --- | --- | --- |
| **Inflammation** | |  | |  | |  |
| Tumor Necrosis Factor, Alpha | | *TNF* | | Increased in adipose tissue of subjects with obesity, induces IR, stimulates inflammatory signaling, induces apoptosis, stimulates and is a target of NFKB-pathway, induces lipolysis in adipocytes | | (1-4) |
| Nuclear Factor Of Kappa Light Polypeptide Gene Enhancer In B-Cells 1 | | *NFKB1* | | Part of NFKB complex which is activated in adipose tissue of subjects with obesity, triggers inflammatory response and the expression of its target genes (*TNF*, *IL6*, *IL1B*), associates with the development of IR in obesity | | (1,3,4) |
| Toll-Like Receptor 4 | | *TLR4* | | Mediates inflammatory signaling, increased in adipose tissue of subjects with obesity, associates with the development of IR, activates NFKB- signaling pathway | | (4,5) |
| Interleukin 6 | | *IL6* | | Increased in adipose tissue of subjects with obesity, induces IR, a target of NFKB-pathway | | (2-4) |
| Interleukin 1, Beta | | *IL1B* | | Increased in adipose tissue of subjects with obesity, induces impaired insulin signaling and IR, induced by the inflammasome complex, target of NFKB-pathway | | (4-6) |
| Interleukin 1 Receptor, Type I | | *IL1R1* | | Mediates IL1B-induced inflammatory signaling, activates NFKB- signaling pathway | | (1) |
| Matrix Metallopeptidase 9 | | *MMP9* | | Increased in adipose tissue of subjects with obesity, remodeling of adipose tissue in obesity, associates with development of IR, possible target of NFKB-pathway, vasculature remodeling | | (4,7) |
| C-Reactive Protein, Pentraxin-Related | | *CRP* | | Increased in adipose tissue of subjects with obesity, marker of systemic inflammation, associates with IR, activates NFKB- signaling pathway | | (4,5) |
| Chemokine (C-C Motif) Ligand 2 | | *CCL2* | | Increased in adipose tissue of subjects with obesity and IR, chemokine for macrophage recruitment in adipose tissue, associates with IR and chronic inflammation | | (2,5) |
| Complement Component 3 | | *C3* | | Increased in adipose tissue of subjects with obesity, pro-inflammatory biomarker for IR in obesity, associates with T2D | | (4,8,9) |

(1) Sorisky A, Molgat AS, Gagnon A. Macrophage-induced adipose tissue dysfunction and the preadipocyte: should I stay (and differentiate) or should I go? Adv Nutr 2013 Jan 1;4(1):67-75.

(2) Greenberg AS, Obin MS. Obesity and the role of adipose tissue in inflammation and metabolism. Am J Clin Nutr 2006 Feb;83(2):461S-465S.

(3) Chung JJ, Markiewicz MA, Polic B, Shaw AS. Role of NKG2D in obesity-induced adipose tissue inflammation and insulin resistance. PLoS One 2014 Oct 15;9(10):e110108.

(4) Berg AH, Scherer PE. Adipose tissue, inflammation, and cardiovascular disease. Circ Res 2005 May 13;96(9):939-949.

(5) Calder PC, Ahluwalia N, Brouns F, Buetler T, Clement K, Cunningham K, et al. Dietary factors and low-grade inflammation in relation to overweight and obesity. Br J Nutr 2011 Dec;106 Suppl 3:S5-78.

(6) Deng T, Cui J. Inflammasomes and Obesity. In: Dannenberg A, Berger N, editors. Obesity, Inflammation and Cancer. Energy Balance and Cancer 7 ed. New York: Springer Science+Business Media New York; 2013. p. 25-60.

(7) Madec S, Chiarugi M, Santini E, Rossi C, Miccoli P, Ferrannini E, et al. Pattern of expression of inflammatory markers in adipose tissue of untreated hypertensive patients. J Hypertens 2010 Jul;28(7):1459-1465.

(8) Engstrom G, Hedblad B, Eriksson KF, Janzon L, Lindgarde F. Complement C3 is a risk factor for the development of diabetes: a population-based cohort study. Diabetes 2005 Feb;54(2):570-575.

(9) Al Haj Ahmad R, Al-Domi H. Complement 3 serum levels as a pro-inflammatory biomarker for insulin resistance in obesity. Diabetes & Metabolic syndrome 2017 Nov;11 Suppl 1:S229-S232.

**Supplementary Table S3.** Spearman’s correlations coefficients between adipose tissue inflammatory score and polyunsaturated fatty acid (PUFA) composition and estimated desaturase indexes in plasma phospholipid (PL) fraction adjusted for age in the FADSDIET study according to the *FADS1* variant (rs174550). SFA, saturated fatty acid; MUFA, monounsaturated fatty acid; LA, linoleic acid; GLA, gamma-linolenic acid; DGLA, dihomo-gamma-linolenic acid; AA, arachidonic acid; D6D, delta-6 desaturase; D5D, delta-5 desaturase; wk, week.

|  |  | **Inflammatory score in adipose tissue** | | | |
| --- | --- | --- | --- | --- | --- |
|  |  | **TT (n=25)** | | **CC (n=28)** | |
| **FAs in serum PL fraction** | **Spearman's correlation** | **0 wk** | **4 wk** | **0 wk** | **4 wk** |
| *SFA* | Correlation Coefficient | **-0.422** | -0.064 | -0.241 | -0.038 |
|  | Sig. (2-tailed) | **0.040** | 0.765 | 0.225 | 0.851 |
| 14:0 | Correlation Coefficient | -0.187 | -0.073 | -0.298 | -0.076 |
|  | Sig. (2-tailed) | 0.382 | 0.736 | 0.131 | 0.707 |
| 16:0 | Correlation Coefficient | -0.273 | 0.012 | -0.041 | -0.025 |
|  | Sig. (2-tailed) | 0.196 | 0.955 | 0.838 | 0.902 |
| 18:0 | Correlation Coefficient | 0.030 | 0.030 | -0.305 | 0.017 |
|  | Sig. (2-tailed) | 0.891 | 0.888 | 0.122 | 0.933 |
| *MUFA* | Correlation Coefficient | -0.162 | **-0.415** | 0.097 | -0.007 |
|  | Sig. (2-tailed) | 0.451 | **0.044** | 0.629 | 0.971 |
| 16:1n-7 | Correlation Coefficient | -0.192 | -0.066 | **-0.415** | -0.249 |
|  | Sig. (2-tailed) | 0.370 | 0.760 | **0.031** | 0.210 |
| 18:1n-9 | Correlation Coefficient | **-0.415** | -0.326 | -0.078 | -0.121 |
|  | Sig. (2-tailed) | **0.044** | 0.120 | 0.699 | 0.548 |
| 18:1n-7 | Correlation Coefficient | 0.113 | -0.219 | 0.118 | 0.356 |
|  | Sig. (2-tailed) | 0.598 | 0.304 | 0.559 | 0.068 |
| 20:1n-9+11 | Correlation Coefficient | **0.512** | -0.278 | 0.236 | 0.078 |
|  | Sig. (2-tailed) | **0.010** | 0.189 | 0.236 | 0.699 |
| *PUFA* | Correlation Coefficient | 0.373 | 0.376 | 0.152 | 0.034 |
|  | Sig. (2-tailed) | 0.073 | 0.070 | 0.450 | 0.865 |
| *n-6* | Correlation Coefficient | **-0.449** | **0.494** | 0.205 | -0.118 |
|  | Sig. (2-tailed) | **0.028** | **0.014** | 0.304 | 0.559 |
| 18:2n-6 (LA) | Correlation Coefficient | -0.170 | **0.544** | 0.167 | -0.204 |
|  | Sig. (2-tailed) | 0.426 | **0.006** | 0.405 | 0.307 |
| 18:3n-6 (GLA) | Correlation Coefficient | N/A | N/A | N/A | N/A |
|  | Sig. (2-tailed) | N/A | N/A | N/A | N/A |
| 20:3n-6 (DGLA) | Correlation Coefficient | -0.355 | 0.074 | -0.212 | -0.057 |
|  | Sig. (2-tailed) | 0.089 | 0.731 | 0.288 | 0.779 |
| 20:4n-6 (AA) | Correlation Coefficient | -0.053 | -0.285 | 0.048 | 0.226 |
|  | Sig. (2-tailed) | 0.806 | 0.177 | 0.812 | 0.257 |
| 22:4n-6 | Correlation Coefficient | **-0.436** | -0.068 | -0.212 | 0.074 |
|  | Sig. (2-tailed) | **0.033** | 0.752 | 0.289 | 0.712 |
| 22:5n-6 | Correlation Coefficient | -0.221 | -0.364 | -0.105 | 0.054 |
|  | Sig. (2-tailed) | 0.299 | 0.081 | 0.601 | 0.789 |
| *n-3* | Correlation Coefficient | **0.589** | -0.199 | -0.066 | 0.130 |
|  | Sig. (2-tailed) | **0.002** | 0.351 | 0.745 | 0.519 |
| 18:3n-3 | Correlation Coefficient | 0.402 | -0.196 | 0.184 | -0.201 |
|  | Sig. (2-tailed) | 0.051 | 0.358 | 0.357 | 0.316 |
| 20:5n-3 | Correlation Coefficient | **0.517** | -0.198 | -0.163 | 0.110 |
|  | Sig. (2-tailed) | **0.010** | 0.353 | 0.418 | 0.585 |
| 22:5n-3 | Correlation Coefficient | -0.155 | -0.395 | -0.133 | 0.030 |
|  | Sig. (2-tailed) | 0.471 | 0.056 | 0.507 | 0.883 |
| 22:6n-3 | Correlation Coefficient | **0.524** | -0.127 | -0.047 | 0.141 |
|  | Sig. (2-tailed) | **0.009** | 0.553 | 0.815 | 0.484 |
| D6D | Correlation Coefficient | -0.236 | -0.161 | -0.239 | 0.039 |
|  | Sig. (2-tailed) | 0.266 | 0.452 | 0.230 | 0.846 |
| D5D | Correlation Coefficient | 0.294 | -0.246 | 0.222 | 0.197 |
|  | Sig. (2-tailed) | 0.164 | 0.247 | 0.266 | 0.324 |

bold values are statistically significant

**Supplementary Table S4.** Spearman’s correlations coefficients between adipose tissue inflammatory score and plasma n-6 polyunsaturated fatty acid (PUFA)-derived eicosanoid concentrations of the FADSDIET study according to the *FADS1* variant (rs174550). LA, linoleic acid; AA, arachidonic acid, LOX, lipoxygenases; COX, cyclooxygenase; CYP, cytochrome P450; sEH, Soluble epoxide hydrolase.

|  |  |  | **Inflammatory score in adipose tissue** | | | |
| --- | --- | --- | --- | --- | --- | --- |
|  |  |  | **TT (n=25)** | | **CC (n=28)** | |
| **Fatty acid precursor** | **Eicosanoid** | **Signaling pathway** | **Baseline^1^** | **Follow-up^1^** | **Baseline^1^** | **Follow-up^1^** |
| LA | *All LA-derived* |  | -0.013 | **0.153**** | 0.096 | -0.070 |
| LA | 13-HODE | LOX | -0.035 | 0.222 | 0.307 | -0.097 |
| LA | 13-oxoODE | LOX | 0.017 | 0.268 | 0.020 | -0.284 |
| LA | 9-HODE | LOX | -0.036 | 0.243 | 0.298 | -0.316 |
| LA | 9-oxoODE | LOX | -0.260 | 0.001 | **0.431*** | -0.091 |
| LA | 9,12,13-TriHOME | LOX | 0.106 | -0.015 | 0.152 | 0.129 |
| LA | 9,10,13-TriHOME | LOX | -0.214 | -0.129 | 0.338 | 0.159 |
| LA | 9,10-EpOME | CYP | 0.309 | 0.123 | 0.008 | -0.013 |
| LA | 12,13-EpOME | CYP | 0.074 | 0.250 | -0.175 | -0.002 |
| LA | 9,10-DiHOME | CYP/sEH | -0.119 | 0.279 | -0.129 | -0.139 |
| LA | 12,13-DiHOME | CYP/sEH | -0.051 | 0.374 | -0.105 | -0.158 |
| LA | EKODE | Auto-ox | 0.061 | 0.069 | -0.091 | 0.037 |
| AA | *All AA-derived* |  | -0.074 | **0.081**** | **0.116**** | **-0.138*** |
| AA | 12-HETE | LOX | -0.072 | 0.027 | 0.192 | -0.188 |
| AA | 15-HETE | LOX | -0.109 | 0.076 | 0.283 | -0.009 |
| AA | 11-HETE | LOX | -0.110 | 0.064 | 0.378 | -0.349 |
| AA | 8-HETE | LOX | -0.397 | 0.035 | 0.005 | 0.125 |
| AA | 5-HETE | LOX | -0.250 | 0.151 | 0.052 | 0.087 |
| AA | 12-KETE | LOX | -0.029 | 0.018 | -0.005 | **-0.564**** |
| AA | 15-KETE | LOX | 0.143 | 0.070 | 0.127 | -0.274 |
| AA | 5-KETE | LOX | -0.092 | -0.256 | -0.004 | -0.203 |
| AA | TXB2 | COX | 0.036 | 0.052 | 0.367 | **-0.420*** |
| AA | PGE2 | COX | 0.013 | 0.114 | 0.295 | -0.217 |
| AA | PGF2a | COX | -0.026 | 0.154 | 0.136 | **-0.430*** |
| AA | 12-HHTrE | COX | -0.010 | 0.076 | 0.339 | **-0.439*** |
| AA | 11,12-EET | CYP | 0.122 | 0.050 | 0.342 | 0.015 |
| AA | 14,15-EET | CYP | 0.228 | 0.047 | 0.063 | 0.018 |
| AA | 8,9-EET | CYP | -0.164 | 0.182 | 0.032 | -0.378 |
| AA | 19-HETE | CYP | -0.344 | 0.131 | 0.075 | 0.151 |
| AA | 5,6-DHET | CYP/sEH | 0.158 | 0.142 | -0.039 | 0.129 |
| AA | 11,12-DHET | CYP/sEH | -0.126 | 0.246 | 0.047 | 0.043 |
| AA | 14,15-DHET | CYP/sEH | -0.312 | 0.258 | -0.035 | 0.010 |
| AA | 8,9-DHET | CYP/sEH | -0.057 | 0.250 | -0.104 | 0.051 |
| AA | 5-iPF2a-VI | Auto-ox | -0.162 | -0.194 | -0.118 | -0.054 |
